# Melatonin Aids in Treating Mood and Sleep Problems Resulting from Hormonal Therapy in Breast Cancer Patients: A Randomized, Double-Blinded, Placebo-Controlled Trial

**DOI:** 10.5812/ijpr-156581

**Published:** 2025-01-06

**Authors:** Nima Vaziri, Melika Shakourifar, Parinaz Sattari, Aliereza Sadeghi, Mehran Sharifi, Ayda Moghadas, Azadeh Moghaddas

**Affiliations:** 1 Department of Clinical Pharmacy and Pharmacy Practice, School of Pharmacy and Pharmaceutical Sciences, Isfahan University of Medical Sciences, Isfahan, Iran; 2 Department of Internal Medicine, Cancer Prevention Research Center, School of Medicine, Isfahan University of Medical Sciences, Isfahan, Iran; 3 Department of Internal Medicine, School of Medicine, Tehran University of Medical Sciences, Isfahan, Iran; 4 Department of Clinical Pharmacy and Pharmacy Practice, Faculty of Pharmacy, Isfahan University of Medical Sciences, Isfahan, Iran

**Keywords:** Breast Cancer, Hormone Therapy, Melatonin, Psycho-Oncology, Dyssomnias, Depression, Mood Disorder

## Abstract

**Background:**

Hormone therapy is commonly used to treat breast cancer but can cause mood disorders and sleep disturbances, negatively impacting patients' well-being.

**Objectives:**

This trial aimed to evaluate the effects of melatonin on sleep problems and mood changes in breast cancer patients undergoing hormone therapy.

**Methods:**

The study was conducted at Omid Hospital in Isfahan, Iran, using a randomized, double-blinded, placebo-controlled design. Participants were assessed using the Hospital Anxiety and Depression Scale (HADS) and were randomly assigned to receive either 6 mg of melatonin or a placebo daily for 4 weeks. Sleep quality, depression levels, and mood states were measured using the Pittsburgh Sleep Quality Index (PSQI), the Center for Epidemiological Studies-Depression Scale (CES-D), and the Profile of Mood States (POMS) Questionnaires at the beginning and end of the 4-week follow-ups.

**Results:**

Sixty participants (34 in the melatonin group and 26 in the placebo group) completed the study. Melatonin administration significantly improved sleep quality, latency, duration, and reduced the use of sleep-promoting medication, according to the PSQI scores. However, there were no significant improvements in depression severity or mood disorders, as assessed by the CES-D and POMS questionnaires, in either group following the 4-week melatonin supplementation period.

**Conclusions:**

Melatonin supplementation effectively alleviated sleep disturbances caused by hormone therapy in breast cancer patients. However, the study did not find substantial evidence supporting the use of melatonin for improving mood disorders or depression in this specific context.

## 1. Background

Cancer encompasses a diverse range of diseases, with more than 100 variations, characterized by uncontrolled cellular proliferation and the acquisition of metastatic properties (1, 2). Among these, breast cancer is the most prevalent life-threatening cancer affecting women worldwide and ranks as the second leading cause of cancer-related mortality, following lung cancer (3). In 2020, it was estimated that approximately 279,100 individuals would be diagnosed with breast cancer, with an estimated 42,690 succumbing to the illness (4). Common treatment modalities for breast cancer include chemotherapy, surgery, radiotherapy, and hormone therapy (5).

Hormone therapy, pioneered by Beatson in 1895, plays a vital role in managing patients diagnosed with estrogen receptor-positive (ER+) breast cancer (6). Hormone therapy commonly employs selective estrogen receptor modulators (SERMs), luteinizing hormone-releasing hormone (LH-RH) agonists, and aromatase inhibitors (AIs) (7, 8). Nonetheless, these medications can elicit adverse effects, including mood disorders, vaginal bleeding, cessation of menstrual cycles, flushing, and depression, due to hormonal fluctuations (9). To address these undesired effects, drugs such as serotonin reuptake inhibitors (e.g., venlafaxine, sertraline, paroxetine) and anticonvulsants (e.g., gabapentin, pregabalin) have been employed (10-15).

Melatonin, a naturally occurring hormone produced by the pineal gland, plays a crucial role in regulating numerous physiological processes in the human body, including mood, sleep, sexual behavior, and circadian rhythm (16, 17). Emerging scientific investigations have shed light on the promising therapeutic implications of melatonin in facilitating the recuperation of individuals diagnosed with breast cancer. Research has demonstrated that melatonin exhibits immunostimulatory effects and enhances the synthesis of interleukin substances, thus positioning it as a viable candidate for therapeutic intervention in breast cancer cases (18, 19). Melatonin also affects estrogen receptor-positive cancer cells, inhibiting cell division and modulating oxidative stress and calcium flow (20).

The menopause transition is frequently associated with sleep disturbances, often attributed to the decline in endogenous melatonin levels. Studies have demonstrated that exogenous melatonin or melatonin analogs have the potential to enhance sleep quality in menopausal women (21). The presence of sleep problems significantly affects overall quality of life, and the use of anti-estrogen endocrine therapies has been identified as a contributing factor to sleep disturbances (22-24).

Extensive research has been conducted on the use of melatonin in individuals diagnosed with cancer, both as a standalone treatment and in combination with chemotherapy. These investigations have consistently reported no significant occurrence of adverse events across various doses and durations (25, 26). Notably, melatonin has shown notable effectiveness in enhancing sleep quality in conditions such as delayed sleep phase disorder, jet lag, and primary insomnia (27-29). Melatonin supplementation has also been found to reduce depressive symptoms, pain, fatigue, and chemotherapy side effects (30, 31).

## 2. Objectives

Considering the prevalent complaints of sleep and mood disorders among women undergoing hormone therapy for breast cancer and the established efficacy of melatonin in managing these symptoms, the objective of this study was to assess the effectiveness of melatonin in treating sleep and mood disorders in breast cancer patients receiving hormone therapy.

## 3. Methods

### 3.1. Participants and Trial Design

The study design for this research involved a randomized, double-blinded, placebo-controlled clinical trial. Participants were recruited between July 2020 and March 2021 at the esteemed hematology-oncology clinic of Omid Hospital, located in Isfahan, Iran. Omid Hospital is a recognized 200-bed facility that specializes in providing comprehensive care for hematology-oncology patients. The ethical considerations of the study protocol were approved by the Isfahan University of Medical Sciences ethics committee (ID number: IR.MUI.RESEARCH.REC.1398.724). Additionally, the trial was officially registered with the Iranian Clinical Trial Registry Center (IRCT20180722040556N3). Before participation, all individuals were provided with informed consent forms, which were reviewed and signed after any uncertainties were thoroughly clarified.

Our research included adult females (over the age of 18) diagnosed with hormone receptor-positive breast cancer who expressed concerns regarding the psychological effects of hormone therapy, including changes in mood and sleep patterns. The participants selected for this study were undergoing anti-hormone therapy using SERMs (e.g., Tamoxifen and Raloxifene) or AIs (e.g., Letrozole), without the concurrent use of chemotherapy or radiotherapy. Prior to the recruitment process, all potential participants were screened using the Hospital Anxiety and Depression Scale (HADS), which had been appropriately translated and validated in Persian by Montazeri et al. (32).

The HADS is a self-reported questionnaire consisting of fourteen items that assess psychological distress in cancer patients, with each question rated on a scale from zero to three. To avoid enrolling patients with severe psychological distress requiring psychiatric intervention, we included only individuals who scored less than 11 on the initial HADS assessment and reported experiencing mood or sleep changes in the past month. Furthermore, participants were required to be capable and willing to adhere to the prescribed oral melatonin regimen.

The exclusion criteria for this study were as follows: Patients with metastatic breast cancer or a previous history of cancer other than breast cancer; patients currently using medications such as propranolol (due to its impact on the central nervous system) or warfarin (due to the potential for harmful drug interactions and adverse effects); patients who did not meet the diagnostic criteria for depression, anxiety disorders, or mood disorders as outlined in the diagnostic and statistical manual of mental disorders (DSM-5); patients who did not require pharmacological treatment for their mood symptoms; patients receiving concurrent anticonvulsant medications for active seizures; patients taking supplements (herbal or non-herbal) that alleviate menopausal symptoms (e.g., soy supplements, vitamin E, clonidine); patients using pharmaceuticals that impact the functioning of the central nervous system for psychiatric conditions (e.g., antipsychotics, antidepressants, anti-anxiety drugs); patients exhibiting any level of liver or kidney impairment; patients diagnosed with autoimmune disorders; patients engaging in smoking, substance abuse, or alcohol consumption; patients with a confirmed hypersensitivity to melatonin-containing products; and patients currently prescribed hypnotic medications, such as benzodiazepines or non-benzodiazepine sleep aids.

### 3.2. Concealment and Randomization

This study, which followed a double-blind design, employed the block randomization technique to ensure randomness. Block randomization software was used to assign unique codes, generated by computer-generated random numbers, to each group (intervention and placebo) (33).

These codes were determined based on statistical factors such as sample size, number of groups, and block size. The 3 mg melatonin tablets were purchased from Razak^®^ Pharmaceutical Company in Tehran, Iran. The indistinguishable placebo tablets were formulated with identical ingredients and excipients (including lactose, microcrystalline cellulose, and magnesium stearate), except for the active melatonin ingredient. These were prepared by the Department of Pharmaceutical Sciences at Isfahan University of Medical Sciences, Isfahan, Iran. The placebo tablets were manufactured to mirror the melatonin tablets in terms of shape, size, color, and packaging. Each package contained 60 tablets and was labeled with the assigned code series by the study's principal investigator in sealed, opaque, sequentially numbered envelopes (SNOSE). Each package contained the group assignment and was opened only after the participant’s details were recorded. None of the participants, investigators, physicians, or statistical analysts were aware of the specific codes assigned to the patients. The allocation process was regularly monitored to ensure adherence to the protocol, and periodic audits were conducted to verify the integrity of the concealment process. Participants were randomly assigned to receive either 6 mg (3 mg twice a day) melatonin or an identical placebo daily for 4 weeks.

### 3.3. Questionnaires

All participants were asked to complete the designated questionnaires at the beginning of the study and again at the completion of the 4-week follow-up period. The questionnaires included the Center for Epidemiological Studies-Depression Scale (CES-D) (34), the profile of mood states (POMS) (35), and the Pittsburgh Sleep Quality Index (PSQI) (36). The obtained results were then compared to the baseline measurements.

The CES-D is a brief self-report scale consisting of 20 questions designed to assess symptoms associated with depression across six distinct scales: Depressed mood, guilt and worthlessness, helplessness and hopelessness, psychomotor retardation, loss of appetite, and sleep disturbances experienced within the past week (34).

The POMS is a clinical psychological rating scale that uses a 40-item questionnaire to evaluate transient mood states. Respondents rate their experiences on a scale from zero (not at all) to five (extremely) (35). The POMS measures six dimensions of mood fluctuations: Tension or anxiety, anger or hostility, vigor or activity, fatigue or inertia, depression or dejection, and confusion or bewilderment (36).

Lastly, the PSQI was used as the final assessed questionnaire. It comprises ten questions focusing on sleep quality, based on the patient's experiences over the past month. This self-administered tool includes a combination of open-ended and event-frequency questions, as well as semantic scales. The PSQI evaluates seven domains: Subjective sleep quality, sleep latency, sleep duration, habitual sleep efficiency, sleep disturbances, use of sleep-promoting medication, and daytime dysfunction (37).

To ensure the validity of the Persian version of these questionnaires, a panel of specialists—including psychologists, a literature expert, oncologists, general physicians, and pharmacists—reviewed the standard criteria. The questionnaires were then translated using a forward-backward method, and both the intervention and control groups, consisting of 34 and 26 patients, respectively, completed the questionnaires four weeks apart. The reliability of the questionnaires was assessed using the intraclass correlation coefficient (ICC).

### 3.4. Patients’ Adherence and Adverse Drug Reactions

At the beginning of the trial, participants received training on how to properly take the provided pills. They were instructed to keep the medication packaging and return it to the investigators at the end of the study. Adherence was monitored using the pill count method. Additionally, participants were asked to maintain a daily diary, recording the time they took their medication and any side effects they experienced. During each follow-up visit, pill count data were recorded, and regular feedback was provided to participants regarding their adherence. Patients who did not adhere to consuming at least 80% of the total cumulative doses of melatonin or the identical placebo were considered non-compliant and excluded from the study. Any reported adverse drug reactions were evaluated according to the common terminology criteria for adverse events (CTCAE) version 4 during the 4-week follow-up period (38, 39).

### 3.5. Statistical Analyses

#### 3.5.1. Sample Size Calculation

We applied the following equation to calculate the sample size for this study and analyze the variations of a particular parameter under two different conditions (40).


$$N=\;\frac{{\left(z_{1}+z_{2}\right)}^{2}[p_{1}\left(1-p_{1}\right)+p_{2}\left(1-p_{2}\right)]}{d^{2}}$$

This equation involves variables such as N (representing the sample size), z_1_ (the reliability coefficient at a 95% level), z_2_ (the power factor at an 80% level), p_1_ and p_2_ (the probability of β power in the first and second groups at 50% each), and d (the study error).


$$N=\;\frac{{\left(1.96+0.84\right)}^{2}[0.5\left(0.5\right)+0.5\left(0.5\right)]}{{\left(0.35\right)}^{2}}≅32$$

**Figure 1.**
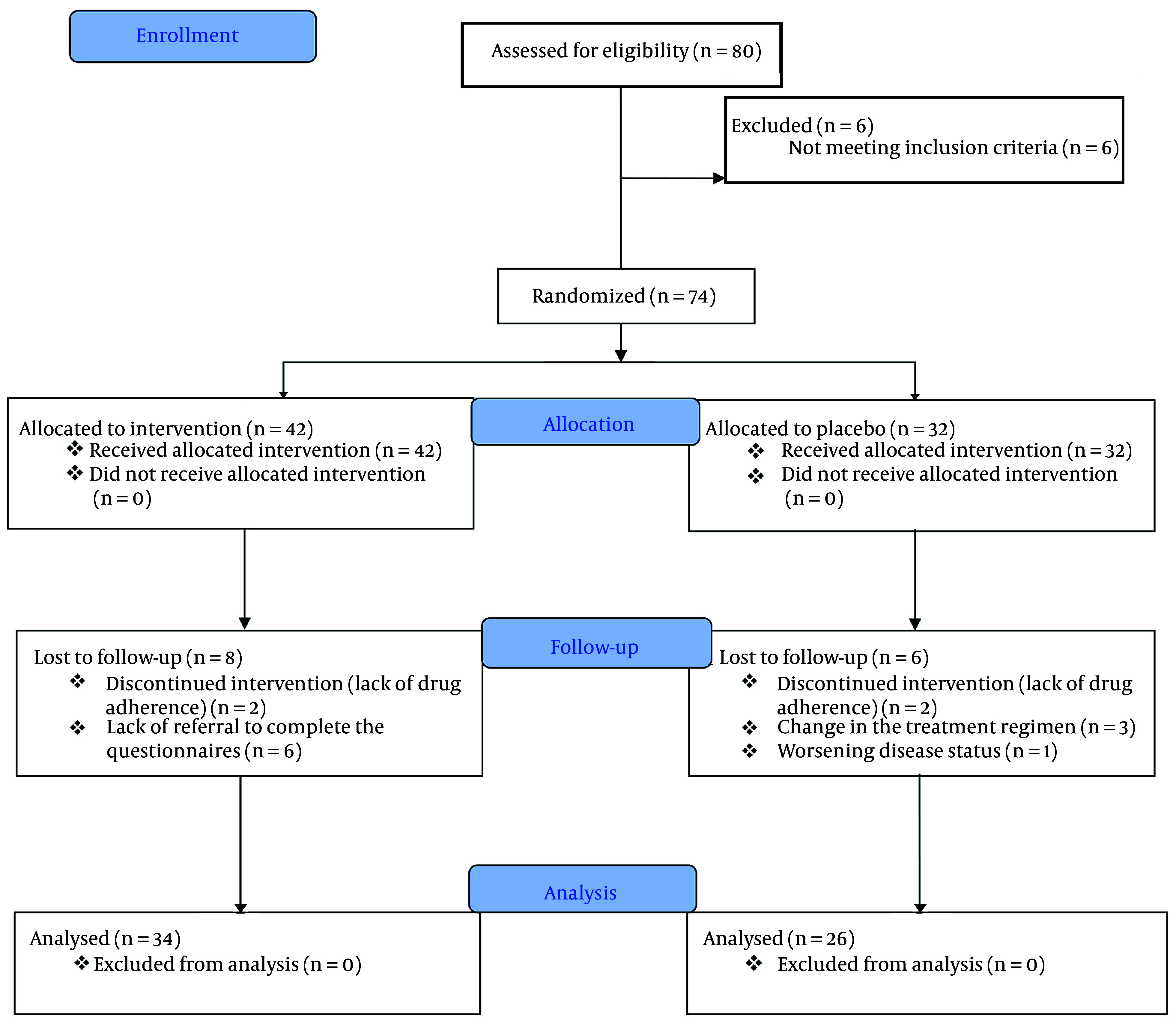
The CONSORT flow diagram of the study

The calculated sample size for each group was anticipated to be 32 individuals. However, due to challenges encountered in identifying eligible patients, the recruitment process was concluded prematurely, resulting in a reduced sample size (refer to the provided flow diagram in Figure 1). 

#### 3.5.2. Statistical Analysis

A per-protocol examination was conducted to compare the data of participants who successfully completed the intervention protocol. The Kolmogorov-Smirnov test was used to assess the normality of data distribution in both the intervention and placebo groups with respect to the demographic and clinical characteristics of the patients. Homogeneity of distribution parameters in the two groups was evaluated using the chi-squared test. Differences between the two groups were assessed using paired *t*-tests, analysis of variance with repeated measures, and Fisher's exact test. A paired-sample *t*-test was used to analyze the mean scores of the CES-D, POMS, and PSQI Questionnaires within a group before and after the intervention. Cronbach's alpha reliability tests were used to measure the internal consistency of the questionnaires.

Data analysis was conducted using the statistical package for the social sciences (SPSS) version 20 software, and statistical significance was determined by a P-value of less than 0.05. For handling missing data, we used complete case analysis, and only complete data from cases were analyzed.

## 4. Results

### 4.1. Clinical and Demographic Characteristics

As shown in Figure 1, a total of 80 eligible participants were enrolled in the research study over the course of one year. Initially, six individuals were excluded due to non-compliance or failure to meet the established criteria, resulting in a final sample of 74 patients who met the criteria and provided informed consent. During the study, six participants from the intervention group were excluded for not completing the required questionnaires. Additionally, two patients were excluded due to non-cooperation and incomplete treatment. In the control group, six participants were excluded due to deteriorating health conditions, changes in treatment plans, and inability to use melatonin. Ultimately, a total of 60 patients successfully completed the treatment protocol and consumed the medication for a duration of 30 days, with 26 individuals in the control group and 34 in the intervention group.

Clinical and demographic data, along with relevant medical information such as age, underlying medical conditions, type of cancer, previous treatments, baseline HADS score, and type of hormone therapy, are shown in Table 1. The mean age was 50.2 ± 8.6 years in the melatonin group and 50 ± 8.7 years in the placebo group. The results indicated that there was no significant difference in terms of other demographic characteristics and variables.

**Table 1.** Baseline Clinical and Demographic Characteristics of Patients in the Intervention and Placebo Groups (N = 60) ^a^

Variables	Intervention Group (N = 34)	Placebo Group (N = 26)	P-Value
**Age (y) **	50.2 ± 8.6	50 ± 8.7	0.91
**Baseline HADS score**	10.2 ± 2.3	10.7 ± 1.8	0.45
**Pathological type of malignancy **			0.60
Ductal	29 (85.29)	23 (88.4)	
Lobular	5 (14.7)	3 (11.5)	
**Family history of cancer**			0.19
Yes	6 (17.6)	8 (35.7)	
No	28 (82.4)	18 (64.3)	
**Comorbidity **			0.48
None	22 (64.7)	19 (73.07)	
Diabetes	0 (0)	2 (7.69)	
Hypertension	7 (20.5)	3 (11.5)	
Hypothyroidism	2 (5.8)	1 (3.4)	
Dyslipidemia	2 (5.8)	1 (3.84)	
**Previous cancer treatments **			0.18
Chemotherapy	9 (28.1)	3 (10.7)	
Radiotherapy	6 (18.8)	4 (14.3)	
Both	17 (53.1)	21 (75)	
**Type of hormone therapy **			0.89
Letrozole	6 (17.6)	2 (7.7)	
Exemestane	3 (8.8)	2 (7.7)	
Tamoxifen	14 (41.1)	14 (53.9)	
Letrozole + Goserelin	5 (14.6)	4 (15.4)	
Tamoxifen + Goserelin	3 (8.8)	2 (7.7)	
Exemestane + Goserelin	3 (8.8)	2 (7.7)	

Abbreviation: HADS, Hospital Anxiety and Depression Scale.

^a^ Values are expressed as mean ± SD or No. (%).

### 4.2. Internal Consistency of Questionnaires

After calculating Cronbach’s alpha as a test score reliability coefficient for the questionnaires, the values were estimated to be 0.94, 0.84, and 0.92 (ranging from 0.6 to 1) for the CES-D, POMS, and PSQI, respectively. These results indicate sufficient reliability and efficiency of the Persian-translated version of the questionnaires.

### 4.3. Results of the Questionnaires

As shown in Table 2, the paired *t*-test analysis revealed no significant differences in the mean scores of the CES-D (P-value = 0.42) and POMS (P-value = 0.15) before and after the intervention in the intervention group. This suggests that the emotional and behavioral states of the patients in the intervention group did not significantly change after the treatment. However, there was a significant improvement in sleep quality (P-value = 0.003), indicating that the intervention led to better sleep quality. In the placebo group, the paired *t*-test results showed that the mean scores of CES-D (P-value = 0.26), POMS (P-value = 0.94), and sleep quality (P-value = 0.10) were not significantly different before and after the intervention.

**Table 2.** The Scores of the Center for Epidemiological Studies-Depression Scale, Profile of Mood States, and Pittsburgh Sleep Quality Index Compared Between the Intervention and Placebo Groups at Baseline and After a 4-Week Follow-up ^a^

Variables (Scores)	Before Intervention	After Intervention	P-Value ^b^
**Intervention group**			
CES-D	20.38 ± 7.91	18.74 ± 8.90	0.42
POMS	50.18 ± 19.09	49.21 ± 18.16	0.15
PSQI	29.18 ± 4.42	5.97 ± 2.73	0.003
Subjective sleep quality	1.26 ± 0.43	1.23 ± 0.43	0.000
Delay in falling asleep	2.00 ± 1.31	2.16 ± 1.42	0.499
Duration of sleep	1.06 ± 0.21	1.02 ± 0.20	0.202
Habitual sleep efficiency	1.51 ± 0.62	1.53 ± 0.63	0.004
Sleep disturbances	0.68 ± 0.28	0.37 ± 0.28	0.000
Use of sleep-promoting medication	0.77 ± 0.96	0.47 ± 0.96	0.000
Daytime dysfunction	0.66 ± 0.69	0.36 ± 0.69	0.683
**Placebo group**			
CES-D	21.00 ± 8.70	18.65 ± 8.89	0.26
POMS	45.96 ± 19.93	45.69 ± 20.85	0.94
PSQI	29.38 ± 4.88	29.08 ± 4.84	0.10
Subjective sleep quality	1.22 ± 0.40	2.04 ± 0.95	0.695
Delay in falling asleep	1.94 ± 1.33	2.47 ± 2.09	0.883
Duration of sleep	1.09 ± 0.23	0.93 ± 0.35	0.609
Habitual sleep efficiency	1.42 ± 0.57	4.19 ± 5.11	0.589
Sleep disturbances	0.60 ± 0.28	2.11 ± 0.28	0.320
Use of sleep-promoting	0.76 ± 0.94	1.81 ± 1.44	0.971
Daytime dysfunction	0.74 ± 0.69	0.44 ± 0.69	0.683

Abbreviations: CES-D, Center for Epidemiological Studies Depression Scale; POMS, profile of mood states; PSQI, Pittsburgh Sleep Quality Index; SD, standard deviation.

^a^ Values are expressed as mean ± SD.

^b^ Data were analyzed by paired *t*-test.

Furthermore, the paired *t*-test analysis demonstrated significant improvements in subjective sleep quality, sleep latency, sleep duration, and the use of sleep-promoting medication after the intervention in the intervention group (P-value < 0.05). However, there were no significant differences in the mean scores for other aspects of sleep quality before and after the intervention (P-value > 0.05). In the placebo group, no significant differences were found in the mean scores for sleep quality before and after the intervention (Table 2). 

As shown in Table 3, an independent *t*-test was conducted to assess the mean scores of CES-D (P-value = 0.51), POMS (P-value = 0.66), and sleep quality before the intervention. Following the intervention, no significant differences were found in the mean scores for CES-D (P-value = 0.97) and POMS (P-value = 0.70) between the two groups. However, a significant difference was observed in sleep quality (P-value = 0.01), with the intervention group showing significantly better sleep quality than the placebo group.

**Table 3.** Comparison Between the Intervention and Placebo Groups in Terms of the Scores on the Center for Epidemiological Studies-Depression Scale, Profile of Mood States, and Pittsburgh Sleep Quality Index at Baseline and After a 4-Week Follow-up ^a^

Variables (Scores)	Intervention Group		Placebo Group		P-Value ^b^ Between Two Groups Before 4 Weeks	P-Value ^c^ Between Two Groups After 4 Weeks
	Baseline	4 Weeks	Baseline	4 Weeks		
**CES-D**	20.38 ± 7.91	18.74 ± 8.90	21.00 ± 8.70	18.65 ± 8.89	0.51	0.97
**POMS**	50.18 ± 19.09	49.21 ± 18.16	45.96 ± 19.93	45.69 ± 20.8	0.66	0.70
**PSQI**	29.18 ± 4.42	5.97 ± 2.73	29.38 ± 4.88	29.08 ± 4.84	0.55	0.01
**Subjective sleep quality**	1.26 ± 0.43	1.23 ± 0.43	1.22 ± 0.40	2.04 ± 0.95	0.43	0.00
**Delay in falling asleep**	2.00 ± 1.31	2.16 ± 1.42	1.94 ± 1.33	2.47 ± 2.09	0.87	0. 07
**Duration of sleep**	1.06 ± 0.21	1.02 ± 0.20	1.09 ± 0.23	0.93 ± 0.35	0.56	0.05
**Habitual sleep efficiency**	1.51 ± 0.62	1.53 ± 0.63	1.42 ± 0.57	4.19 ± 5.11	0.47	0.00
**Sleep disturbances**	0.68 ± 0.28	0.37 ± 0.28	0.60 ± 0.28	2.11 ± 0.28	0.96	0.89
**Use of sleep promoting medication**	0.77 ± 0.96	0.47 ± 0.96	0.76 ± 0.94	1.81 ± 1.44	0.95	0.00
**Daytime dysfunction**	0.66 ± 0.69	0.36 ± 0.69	0.74 ± 0.69	0.44 ± 0.69	0.88	0.88

Abbreviations: CES-D, Center for Epidemiological Studies-Depression Scale; POMS, profile of mood states; PSQI, Pittsburgh Sleep Quality Index; SD, standard deviation.

^a^ Values are expressed as mean ± SD.

^b^ Data were analyzed by independent *t*-test.

^c^ Data were analyzed by Paired *t*-test.

The independent-samples *t*-test revealed no significant differences in the mean scores of any sleep quality disorder subgroups between the two groups before the intervention (P-value > 0.05). However, the paired *t*-test indicated significant differences in the mean scores for subjective sleep quality, sleep latency, sleep duration, and the use of sleep-promoting medication between the two groups after the intervention (P-value < 0.05). Nonetheless, no significant differences were found in the mean scores for other aspects of sleep quality disorder between the two groups.

### 4.4. Adverse Effects

As indicated in Table 4, the analysis based on the CTCAE version 4 (38) revealed no unexpected adverse effects during the 4-week administration of 6 mg of melatonin per day in either the intervention or placebo group. However, it is noteworthy that three cases of mild to moderate excessive daytime sleepiness were reported in the melatonin group. Despite this, the difference between the two groups was not statistically significant (P-value = 0.18).

**Table 4.** The Reported Adverse Drug Reactions in Both the Intervention and Placebo Groups During the 4-Week Follow-up

Adverse Drug Reaction	Intervention Group	Placebo Group	P-Value
**Nausea**	13	13	0.38
Grade 1	7	10	
Grade 2	5	2	
Grade 3	1	1	
**Vomiting**	6	5	0.26
Grade 1	4	3	
Grade 2	1	2	
Grade 3	1	0	
**Daytime sleepiness**	3	0	0.18
Grade 1	2	0	
Grade 2	1	0	

## 5. Discussion

In this randomized controlled trial, the administration of melatonin for a duration of four weeks in patients with hormone-positive breast cancer resulted in a statistically significant improvement in sleep quality, particularly on the subscales of sleep quality, sleep latency, and sleep problems. However, no significant differences were observed in the mean changes of CES-D and POMS scores, indicating no notable mood changes in the intervention group.

Hormone therapy utilizing various classes of drugs is a primary treatment option for breast cancer patients with hormone receptors. However, these drugs may lead to adverse effects such as mood disorders, depression induced by hormone deficiency, sleep disturbances, and hot flashes (6).

Based on the results of our study, taking a 6 mg dose of melatonin for four weeks improved sleep quality and reduced sleep problems, which is consistent with the findings of a study published by Madsen et al. (41) in 2016. This study investigated the effect of melatonin on sleep outcomes in breast cancer patients considered for surgery. In that study, patients were randomly assigned to receive either 6 mg of melatonin or a placebo daily from three nights before surgery to at least one week postoperatively. A significant difference was found between the two groups in terms of improved sleep quality and reduced awakening time during nighttime sleep.

Additionally, the potential impact of melatonin on psychological conditions such as depression, bipolar disorder, and seasonal affective disorder has been recognized by numerous studies (42, 43). Melatonin appears to play an important role in managing mood disorders in patients with severe depression due to its safety and its potential ability to regulate the body's circadian rhythm during depressive episodes (44). Today, agomelatine, a substance known as a selective agonist of melatonin and an antagonist of 5-HT2B and 5-HT2C receptors, has been identified as a chronobiotic, antidepressant, and anxiolytic agent (43). In the short term, it exhibits antidepressant activity similar to that of venlafaxine, fluoxetine, and sertraline (44).

In a recent study conducted by Wang et al. in 2021, it was found that melatonin has the ability to alleviate anxiety-like behaviors induced by sleep deprivation. This effect is achieved through the amelioration of oxidative stress, neuroinflammation, activation of the NF-kB pathway, apoptosis, and autophagy (45).

Additionally, in a study conducted by Chen et al. (42) in 2014, the impact of melatonin on sleep and mood disorders in breast cancer patients was investigated. The participants were randomly assigned to receive either a daily dosage of 3 mg of melatonin or a placebo for a duration of four months. The results revealed a significant difference between the two groups in terms of sleep quality scores (P < 0.001). However, no significant difference was observed between the two groups regarding the improvement of mood disorders. Despite the disparity in melatonin dosage between our study (6 mg daily) and Chen's study (3 mg daily), the findings of our study were consistent with the results of Chen et al. (42).

In a study conducted by Innominato et al. (43) in 2016, the impact of melatonin on enhancing sleep quality in breast cancer patients was explored. Furthermore, a recent study by Palmer et al. (46) in 2020 demonstrated that melatonin not only enhanced sleep quality in breast cancer patients undergoing chemotherapy but also exhibited neuroprotective properties. It was observed to alleviate symptoms of depression in patients who received melatonin. These findings align with the results of our study regarding the enhancement of sleep quality. The positive effects on depression may be attributed to the higher melatonin dosages used in this study (20 mg daily) compared to our own study (6 mg daily).

In recent years, many researchers have investigated the potential effects of melatonin in facilitating the recovery of breast cancer patients, and numerous studies have been conducted to test this theory (20, 47). Clinical evidence suggests that melatonin stimulates the immune system, leading to increased production of interleukin products (18). It also affects estrogen receptors in cancer cells at physiological concentrations (19). Melatonin has been found to reduce cell division by halting the cell cycle, inducing apoptosis, and modulating mitotic activating protein kinase (MAPK) signaling pathways (47). Furthermore, it modulates oxidative stress and calcium flow in cancer cells, which contributes to progress in breast cancer recovery (20).

As mentioned earlier, melatonin plays a prominent role in regulating the sleep cycle. On the other hand, it is well-established that hormone therapy can cause sleep problems, mood swings, and depression as common side effects. Studies have shown that melatonin can effectively treat estrogen deficiency insomnia in postmenopausal women (48). Additionally, it can alleviate sleep problems associated with fibromyalgia syndrome, a condition characterized by tenderness, sensitivity, and muscle weakness (21). Given the potential effects of melatonin on enhancing sleep quality, it is expected to help improve mood as well (42).

Furthermore, a recently published study examined the effects of long-term melatonin use (3 mg in the morning and 5 mg at night for 12 months) in non-cancerous postmenopausal women. The study found that melatonin improved psychosomatic disorders in these women. Despite the lack of impact on serum levels of follicle-stimulating hormone (FSH) and estradiol, melatonin was found to effectively decrease psychosomatic symptoms (49).

In addition to the effectiveness of melatonin, several studies have demonstrated that the administration of 6 mg of melatonin is safe and well-tolerated (50, 51). For example, in a study by Farrokhian et al., 6 mg of melatonin was administered to a type 2 diabetes mellitus population for 8 weeks, and, aside from somnolence, there were no significant or severe adverse drug reactions among the enrolled patients (50).

It is an undeniable fact that the long-term complications resulting from the administration of sex hormone-reducing drugs have a detrimental impact on the well-being of patients, often leading to medical intervention in severe cases. Based on this evidence, it has been successfully demonstrated that the consumption of melatonin over a period of four weeks can enhance the quality of sleep in individuals diagnosed with hormone-positive breast cancer. More specifically, improvements were observed in subscales measuring the mental quality of sleep, sleep duration, habitual sleep efficiency, and the utilization of sleep-promoting medications. No significant differences were observed between the intervention and placebo groups regarding the average changes in CES-D and POMS scores, which indicate mood swings and depression status. However, further studies incorporating distinct designs and specific indicators to evaluate the impact of melatonin on mood disorders and sleep quality in breast cancer patients are imperative in order to comprehensively assess its effects on various psychiatric disorders and overall quality of life.

Despite the promising results, this study has several limitations. First, the sample size was relatively small, which may limit the generalizability of the findings. Second, the study duration was short, and the long-term effects of melatonin on sleep quality and overall health were not assessed. Third, the study relied on self-reported measures of sleep quality, which can be subjective and prone to bias. Future research should include larger, more diverse populations and utilize objective sleep assessment tools to validate these findings.

### 5.1. Conclusions

The findings of the study indicated that the administration of melatonin (6 mg daily for a duration of 4 weeks) can effectively alleviate sleep disturbances caused by hormonal therapy in breast cancer patients. Specifically, improvements were observed in sleep latency, disturbance, and quality, along with a reduction in the use of sleep-promoting medications. Therefore, melatonin can be considered a supplementary treatment option to enhance sleep quality in this patient population. However, it is important to note that melatonin did not yield significant improvements in mood disorders and depression in this particular study. To validate these findings and determine the optimal dosage and duration of melatonin supplementation, larger-scale studies are warranted.

## Data Availability

The dataset presented in the study is available on request from the corresponding author during submission or after publication.

## Clinical Trial Registration Code:

IRCT20180722040556N3.
